# Sudan Ebola virus (SUDV) outbreak in Uganda, 2022: lessons learnt and future priorities for sub-Saharan Africa

**DOI:** 10.1186/s12916-023-02847-1

**Published:** 2023-04-13

**Authors:** Godfrey Bwire, Benn Sartorius, Philippe Guerin, Merawi Aragaw Tegegne, Sam I. Okware, Ambrose O. Talisuna

**Affiliations:** 1 Division of Public Health Emergency Preparedness and Response, Ministry of Health, Kampala, Uganda; 2 Department of Community Health and Behavioural Sciences, Makerere University School of Public Health, Kampala, Uganda; 3 Centre for Tropical Medicine and Global Health, University of Oxford, Oxford, UK; 4 Infectious Diseases Data Observatory (IDDO), Oxford, UK; 5 Division of Emergency Preparedness & Response, Africa Union/Africa CDC, Addis Ababa, Ethiopia; 6 Uganda National Health Research Organization (UNHRO), Entebbe, Uganda; 7 World Health Organization, Liaison Office to the African Union (AU) and the United Nations Economic Commission for Africa (UNECA), Addis Ababa, Ethiopia

**Keywords:** Ebola, Outbreak, Africa, Uganda, Sub-Saharan Africa, Sudan Ebola virus, Viral haemorrhagic fever, Biosecurity, Biosafety, Infection prevention and control

## Background

On 20th September 2022, the Ugandan Ministry of Health (MOH) declared an outbreak of haemorrhagic fever caused by the Sudan Ebola virus (SUDV) following laboratory confirmation of a patient from a village in Madudu sub-county, Mubende district [1]. EVD is highly contagious with a high mortality rate. By the end of the SUDV outbreak in Uganda on 11th January 2023, 164 cases (probable and confirmed) were traced with 55 confirmed deaths [2]. This was the sixth reported SUDV outbreak in Uganda since the first in 2000 where a cumulative total of 325 cases and 224 deaths were recorded. Overall case-fatality ratio (CFR) during the 2022 SUDV was 39% (55/142). Despite its recurrence and high impact, SUDV appears to be neglected in terms of investment in research and development of medical countermeasures. The first documented EVD outbreak occurred in 1976 (47 years ago) in communities located near the Ebola river and was designated Ebola Zaire. In the same year, an Ebola outbreak occurred in Sudan caused by a different virus strain that was designated SUDV. Since then, there have been several SUDV outbreaks, all in sub-Saharan Africa (SSA) [3]. The most deadly of all EVD outbreaks occurred in 2014–2016, in West Africa, where 11,310 deaths and approximately 28,600 cases were recorded [3]. There is no specific treatment or vaccine for SUDV but it exists for the Zaire strain [1]. Consequently, there is an urgent need to address the current SUDV outbreak control gaps.

## Main text

Although the SUDV outbreak in Uganda ended, several key priorities and gaps remain regardings reducing the impact of future SUDV outbreaks in SSA. These include:

1. *Research and development of effective vaccines for SUDV*: Coronavirus disease 2019 (COVID-19) showed the world that with commitment, significant progress can be achieved through the discovery and rapid licensing of a range of effective vaccines within 2 years [4]. Investments for high-threat pathogens such as SUDV are often reactive and follow outbreaks [5]. Funding for SUDV vaccine development, testing and implementation needs to be more proactively guided. This will require collaborations in timely sharing of genome sequences and for the Ministries of Health (MOHs) to work with academia and research institutions to define a clear SUDV research agenda and timelines for achieving deliverables. The Coalition for Epidemic Preparedness Innovations (CEPI) has kickstarted investment for candidate SUDV vaccines that are yet to undergo clinical trials in humans. However, capacity will need to be created for vaccine manufacturing, with a transfer of technology over time in SSA. Hence more investment by CEPI and the African Union (AU) in SUVD vaccine development is urgently required.
2. *Reliable rapid diagnostic tests (RDTs)*: The inability to act rapidly, diagnose and isolate cases in previous EVD outbreaks has been demonstrated to be an important factor in the large-scale progression of outbreaks within the SSA region [6]. The current reliable laboratory tests for EVD take 6–12 h, which is too long given that this is a highly infectious disease. This time lapse allows symptomatic cases to potentially infect other individuals and delays commencement of contact tracing and outbreak containment. With more research on SUDV, it is possible to develop quicker rapid tests as is the case with other prominent pathogens such as COVID-19 and HIV. Of course, there may be technological challenges associated with the development of a reliable SUDV test as is the case with rotavirus, Japanese encephalitis or yellow fever. However, with more investment, an improvement could be made on the current RDTs.
3. *Effective therapeutic for treatment of SUDV cases*: SUDV has a very high CFR. Yet there are no licensed effective therapeutics [1] due to inadequate is research and development investment. The COVID-19 pandemic has demonstrated how rapidly novel therapeutics can be developed when there is sufficient global investment. A lot of the research/investment undertaken in the context of COVID-19 was focused on the needs of wealthy countries but not enough was done to address the specific needs of LMICs [7]. The relative lack of investment in EVD research is glaringly demonstrated because currently there are only 139 registered EVD trials compared to 7551 COVID-19 trials [WHO Clinical Trials Registry Platform (ICTRP)] [8]. Given the pandemic potential, rapid spread and high CFR of SUDV and the high burden to vulnerable countries in SSA, it is essential to enhance investment for novel therapeutics.
4. *Timely support to affected countries to build resilient communities for SUDV outbreaks*: Previous outbreaks have largely occurred and impacted SSA. Given the lower Human Development Indices (HDIs) and smaller economies in SSA, provision of timely resources through regional and international institutions such as the WHO and Africa CDC, PANdemic preparedness latform for Health and Emerging infectious Response (PANTHER) are essential. Investment in tools such as those from the International Severe Acute Respiratory and Emerging Infection Consortium (ISARIC) should be promoted [9]. Establishment of SUDV isolation units requires a lot of resources which are not available in SSA.
5. *Enhanced investment in SUDV research*: There is an urgent need to identify reservoirs and inanimate sources of the virus. Many previous EVD outbreaks have occurred in very remote rural areas and forested communities and some wildlife have been implicated as potential reservoirs, however, the reservoir for SUDV remains unknown [3]. Infections have also occurred around mines calling for the examination of inanimate objects [1]. This knowledge gap remains large. Hence, more studies are needed to identify reservoirs for SUDV to limit future exposure of populations in close proximity.
6. *Improving approaches for contact tracing, infection prevention and control, biosafety and biosecurity*: Rapid contact tracing is key in the prevention of new cases and maximising EVD outbreak containment. The use of technology including the use of digital contact tracing should be promoted during SUDV outbreaks. SUDV is highly infectious and deadly. Therefore, all at-risk personnel and community members should be protected. During the 2022 SUDV outbreak in Uganda, healthcare worker infection accounted for 13.4% (19) of the cases and 12.7% (7) of the deaths [10]. Therefore, investment in infection prevention and control, biosafety and biosecurity should be of paramount importance.
7. *Strengthening risk communication and community engagement*: In the recent outbreak, risk communication and community engagement was critical in early detection, contact tracing and sustenance of prevention and management efforts. However, gaps remain. Despite several outbreaks of EVD in Uganda to date, communities are still misinformed to the extent that some dead bodies were exhumed after safe and dignified burials leading to further infection transmission in the community.
8. *Creating a pool of trained responders at country, regional and continental levels*: During EVD/SUDV outbreaks the local governments are often overwhelmed. However, when there is a trained pool of responders within the country and region, it is possible to reduce the impact of SUDV in affected communities through timely deployment of the trained personnel. We propose that multi-disciplinary teams of responders are established at sub-national, national, regional and continental level ready to be deployed in case of such outbreaks.

## Conclusions

SUDV outbreaks remain a major public health threat in SSA almost five decades since the first outbreak was detected in 1976. Despite recurrent outbreaks of SUDV/EDV, the disease remains neglected with limited investment in research and development of medical countermeasures and other innovations for its rapid containment. We call upon governments, regional bodies, continental and global stakeholders to invest in the eight (8) critical areas we have listed above if the SSA region is to rapidly prevent, mitigate and respond to future SUDV outbreaks.
